# Targeting novel coronavirus SARS-CoV-2 spike protein with phytoconstituents of *Momordica charantia*

**DOI:** 10.1186/s13048-021-00872-3

**Published:** 2021-09-27

**Authors:** Santosh Kumar Singh, Shailesh Singh, Rajesh Singh

**Affiliations:** 1 Department of Microbiology, Biochemistry and Immunology, Morehouse School of Medicine, 720 Westview Drive SW, Atlanta, GA 30310 USA; 2 Cancer Health Equity Institute, Morehouse School of Medicine, 720 Westview Drive SW, Atlanta, GA 30310 USA

**Keywords:** Bitter melon, SARS-CoV-2, SPR, Erythrodiol, Momordica Charantia

## Abstract

**Background:**

Infections by the SARS-CoV-2 virus causing COVID-19 are presently a global emergency. The current vaccination effort may reduce the infection rate, but strain variants are emerging under selection pressure. Thus, there is an urgent need to find drugs that treat COVID-19 and save human lives. Hence, in this study, we identified phytoconstituents of an edible vegetable, Bitter melon (*Momordica charantia*), that affect the SARS-CoV-2 spike protein.

**Methods:**

Components of *Momordica charantia* were tested to identify the compounds that bind to the SARS-CoV-2 spike protein. An MTiOpenScreen web-server was used to perform docking studies. The Lipinski rule was utilized to evaluate potential interactions between the drug and other target molecules. PyMol and Schrodinger software were used to identify the hydrophilic and hydrophobic interactions. Surface plasmon resonance (SPR) was employed to assess the interaction between an extract component (erythrodiol) and the spike protein.

**Results:**

Our *in-silico* evaluations showed that phytoconstituents of *Momordica charantia* have a low binding energy range, -5.82 to -5.97 kcal/mol. A docking study revealed two sets of phytoconstituents that bind at the S1 and S2 domains of SARS-CoV-2. SPR showed that erythrodiol has a strong binding affinity (KD = 1.15 μM) with the S2 spike protein of SARS-CoV-2. Overall, docking, ADME properties, and SPR displayed strong interactions between phytoconstituents and the active site of the SARS-CoV-2 spike protein.

**Conclusion:**

This study reveals that phytoconstituents from bitter melon are potential agents to treat SARS-CoV-2 viral infections due to their binding to spike proteins S1 and S2.

## Background

On January 30, 2020, the World Health Organization (WHO) declared infections of severe acute respiratory syndrome coronavirus 2 (SARS-CoV-2) a global emergency. Originating in Wuhan, China, in December 2019, the disease (COVID-19) took only four months to become a pandemic [1]. It has now spread to more than 221 countries and territories [2]. With a scarcity of treatment options and lack of vaccines, especially in low-income countries, COVID-19 has taken 4 million lives worldwide. This infection involves dosing with nucleoside analogs, such as remdesivir, that inhibit viral replication by becoming incorporated into RNA [3, 4]; others are supporting care, symptoms, experimental measures, and isolation. Repurposing of drugs and administering vaccines are believed to be promising to deal with this pandemic [5]. Given its drastic effects, many countries, including the United States, have employed extensive resources to find a definitive cure for COVID-19 and develop preventive vaccines.

Scientific knowledge of the virus and COVID-19 are accumulating. However, treatment remains a challenge for physicians and patients. In a viral infection, the host–pathogen interaction is a critical step that can be utilized to develop antiviral therapy [6]. Further, cancer patients are more vulnerable to COVID-19 infections and, due to tumor-induced immune suppression, have a high frequency of severe symptoms [7].

Like Middle East respiratory syndrome coronavirus (MERS-CoV), SARS-CoV-2 has crossed the species barrier and has reached humans as deadly pneumonia [8]. Bats are considered the primary host for SARS-CoV-2, with palm civets and raccoon dogs as intermediate hosts [9, 10]. The zoonotic transmission of SARS-CoV-2 between bats and humans remains to be described. SARS- CoV, which caused a previous outbreak of severe acute respiratory syndrome, and SARS-CoV-2 have the same origin, are structurally related with 80% genotypic similarity, and have the same binding affinities to human angiotensin-converting enzyme 2 (ACE2) [8, 11]. Like SARS-CoV, SARS-CoV-2 is a positive-strand virus with surface protein subunits S1 and S2 that bind to ACE2. In SARS-CoV-2, the spike glycoprotein (S protein) is a trimeric protein that is cleaved into S1 and S2 subunits, of which S1 facilitates binding to the receptor, ACE2. S2 is cleaved by host proteases, mediates membrane fusion, and allows the virus to enter into host cells, an essential step in infection [12–15]. Structural studies of SARS-CoV-2 show that docking of the S protein trimer onto the structure of the ACE2 dimer suggests simultaneous binding of two S protein trimers [16]**.** In addition, SARS-CoV-2 has a furin cleavage site between the S1 and S2 subunits that distinguishes SARS-CoV-2 from other SARS-CoVs [8]. Further, scanning electron micrograph studies show that monoclonal antibodies specific to the SARS-CoV receptor-binding domain (RBD) have no appreciable affinity to SARS-CoV-2, suggesting antibody cross-reactivity between the two viruses RBDs [17]. Thus, there is an urgent need to identify strategies that facilitate the development of decoy ligands to neutralize or suppress viral infection.

Natural products and their derivatives have been used to cure several diseases, including viral infections. A library of chemicals is available and needs to be explored to develop drugs to treat viral infections [18]. There are reports pointing to herbal medications and traditional medicine for treating SARS and other viral infections [19]. Among the sources of natural remedies, vegetables and fruits are rich sources of vitamins, dietary fiber, and minerals linked to reducing cardiovascular diseases, cancer, and other chronic diseases [20]. Among the vegetables, *Momordica charantia,* commonly known as bitter melon or bitter gourd, is consumed primarily in China, India, and Pakistan. Bitter melon has many traditional uses, including treatment of nephropathy, neuropathy, gastroparesis, cataracts, and atherosclerosis. They are further inhibiting human immunodeficiency virus (HIV) from the compounds extracted from bitter melon, such as momordica anti-HIV protein (30 kDa, MAP-30) and gelonium anti-HIV protein (31 kDa, GAP-31). These compounds reduce viral infections in a concentration-dependent manner by inhibiting the HIV-1 integrase protein, remaining harmless to uninfected cells and unable to enter healthy cells [21, 22]. Moreover, among several compounds extracted from *Momordica charantia*, erythrodiol displayed potent anti-inflammatory and immunomodulatory activities and activity against HIV-1 reverse transcriptase [23, 24]. Compounds that impede viral replication by binding to viral surface proteins could also exist, and these need to be identified.

In the present investigation, using molecular modeling and docking, we identified a natural compound from a bitter melon that could potentially block binding of the S1 and S2 proteins of CoV-2 and thereby inhibit SARS-CoV-2 infections.

## Results

### Molecular docking of phytoconstituents of bitter melon to SARS-CoV-2 S (spike protein)

**Fig. 1:**
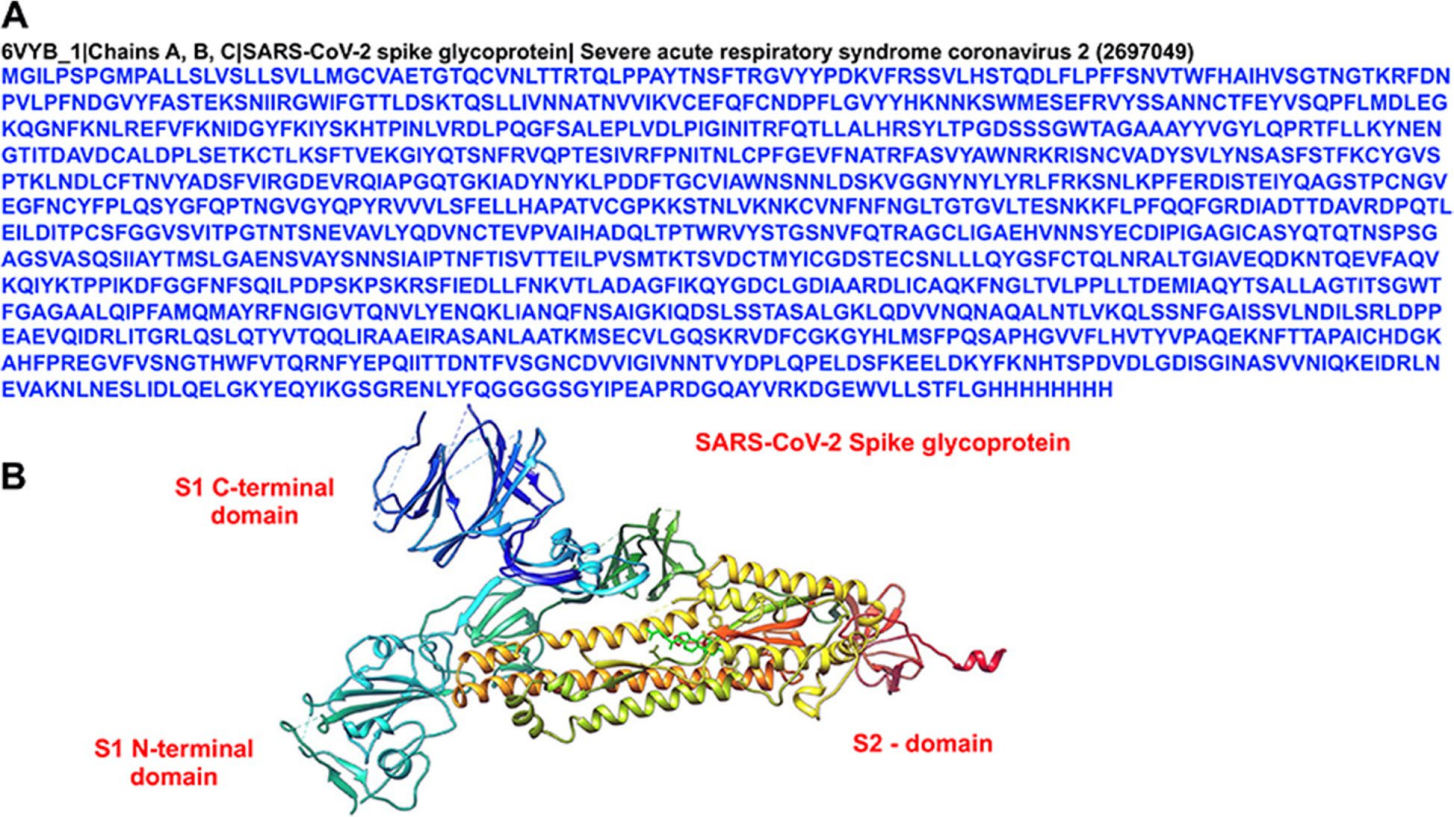
The structure of SARS-CoV-2 spike protein (PBD-ID: 6VYB). **A** FASTA sequence of the SARS-CoV-2 spike protein chain **A,** **B** & **C**. **B** The molecular structure is showing the surface of SARS-CoV-2 spike protein chain A

**Fig. 2:**
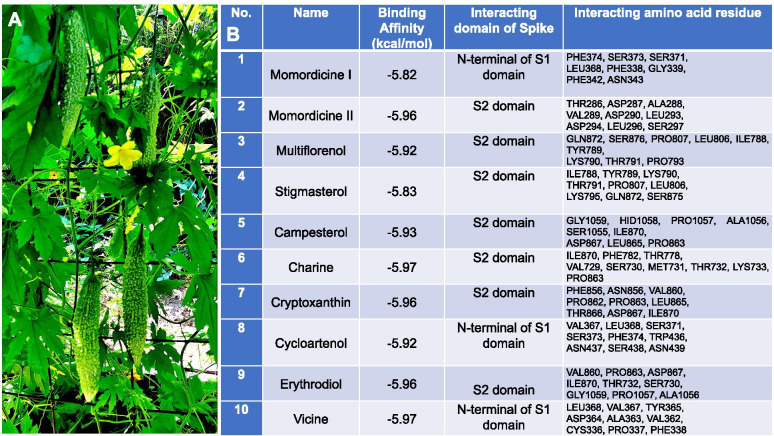
Interaction of phytoconstituents of bitter melon with the domain of spike protein: **A** Bitter melon *(Momordica charantia)* plants. **B** Table shows the docking results of hydrogen bonding interaction between phytoconstituents to the active site of SARS-CoV-2 spike protein S1 and S2

**Fig. 3:**
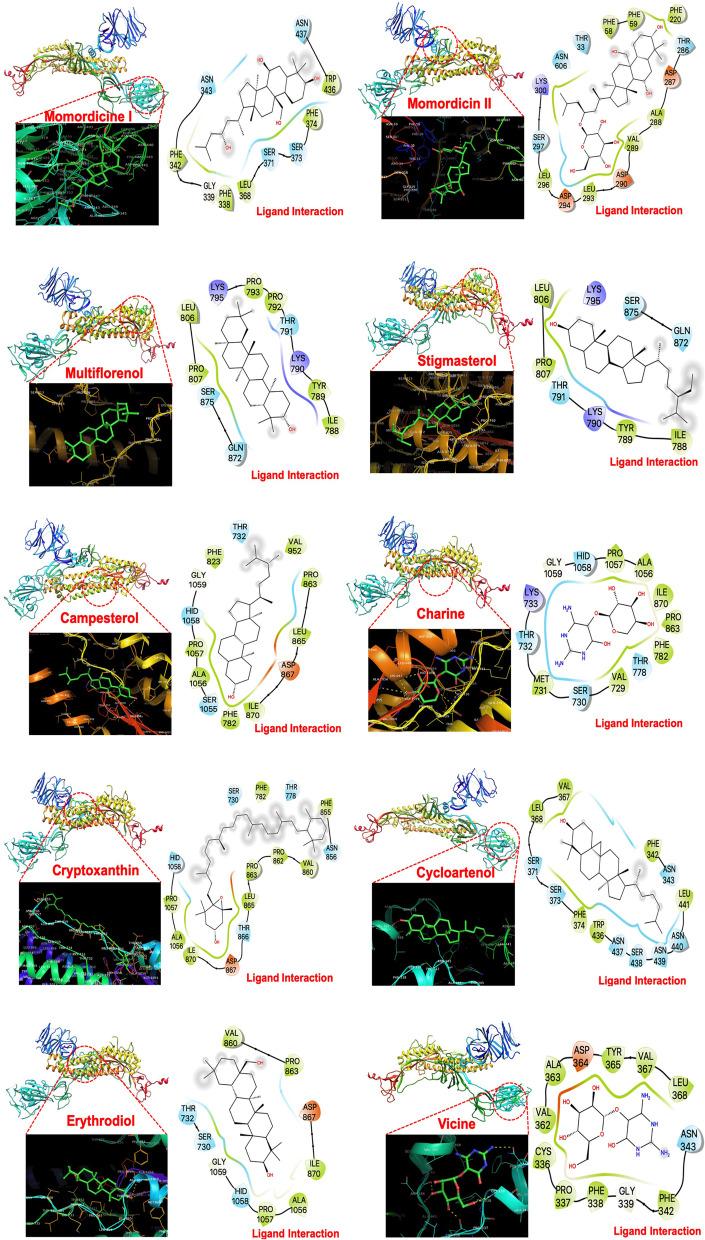
The molecular docking of SARS-CoV-2 spike protein and bitter melon phytoconstituents: The 3-dimensional ribbon structure of SARS-CoV-2 spike protein and stick model of bitter melon phytoconstituents showing the binding site of the SARS-CoV-2 spike protein. The LigPlot structure is showing the interaction with amino acid residues of SARS-CoV-2 spike protein

Binding energy (Kcal/mol) data allow comparisons of the affinities of various ligands/compounds with their corresponding target receptor molecules. Lower binding energy indicates a higher affinity of the ligand for the receptor. The structure of the SARS-CoV-2 spike protein is shown in Fig. 1. The phytoconstituents of bitter melon (*Momordica charantia*) displayed low binding energies in the range of -5.82 to -5.97 kcal/mol and similar binding preferences for the S2 domain of the spike protein (Fig. 2). However, momordicine I, cycloartenol, and vicine are bound to the N-terminal of the S1 domain. Despite having a similar preference for the S2 and S1 domains, an additional hydroxyl group on the chromone ring of various constituents of bitter melon affected its hydrogen-bonding interactions with various residues of the S2 and S1 domains. As shown in Figs. 2 and 3, momordicine I interacted with the S1 spike domain through PHE374, SER373, SER371, LEU368, PHE338, GLY339, PHE342, and ASN343 residues through H-bonding and hydrophobic interactions (based on 3D views of different -OH groups forming H-bonds). Similarly, momordicine-II interacted with THR286, ASP287, ALA288, VAL289, ASP290, LEU293, ASP294, LEU296, and SER297; multiflorenol with GLN872, SER876, PRO807, LEU806, ILE788, TYR789, LYS790, THR791, and PRO793; stigmasterol with ILE788, TYR789, LYS790, THR791, PRO807, LEU806, LYS795, GLN872, and SER875; campesterol with GLY1059, HID1058, PRO1057, ALA1056, SER1055, ILE870, ASP867, LEU865, and PRO863; charine with ILE870, PHE782, THR778, VAL729, SER730, MET731, THR732, LYS733, and PRO863; cryptoxanthin with PHE856, ASN856, VAL860; PRO863, LEU865, THR866, ASP867, and ILE870; cycloartenol with VAL367, LEU368, SER371, SER373, PHE374, TRP436, ASN437, SER438, and ASN439; erythrodiol with VAL860, PRO863, ASP867, ILE870, THR732, SER730, GLY1059, PRO1057, and ALA1056; and vicine with LEU368, VAL367, TYR365, ASP364, ALA363, VAL362, CYS336, PRO337, and PHE338. Overall, the docking study disclosed two distinct sets of ligands that bind at the S1 and S2 domains of the SARS-CoV-2 spike protein.

### Absorption, distribution, metabolism, and excretion (ADME) profiling of the phytoconstituents

The Lipinski rule of five [25, 26], which is often used to evaluate potential interactions between candidate drugs and target molecules, evaluates the propensity of a compound with a notable pharmacological or biological activity to be used as a potential drug. The rule serves as a filter to screen potential therapeutic agents/drugs at the program's initiation, thereby minimizing the labor and cost of exercises involving clinical drug development and, to a large extent, preventing late-stage clinical failures. The rule mainly determines the various molecular properties of a compound that are its prime characteristics to be a potential drug. Lipinski’s rule states that, for any compound to be selected as a potential drug, it should have (a) a molecular mass < 500 daltons, (b) high lipophilicity (expressed as LogP < 5), (c) less than 5 hydrogen bond donors, (d) less than 10 hydrogen bond acceptors, and (e) a molar refractivity between 40–130. If a compound of interest possesses more than two of the abovementioned criteria, it is likely to be a candidate for drug development. The phytochemicals used in this study passed all five of Lipinski’s criteria (Table 1). Thus, we suggest that these phytochemicals have the potential capacity to function effectively as drugs.

**Table 1:**
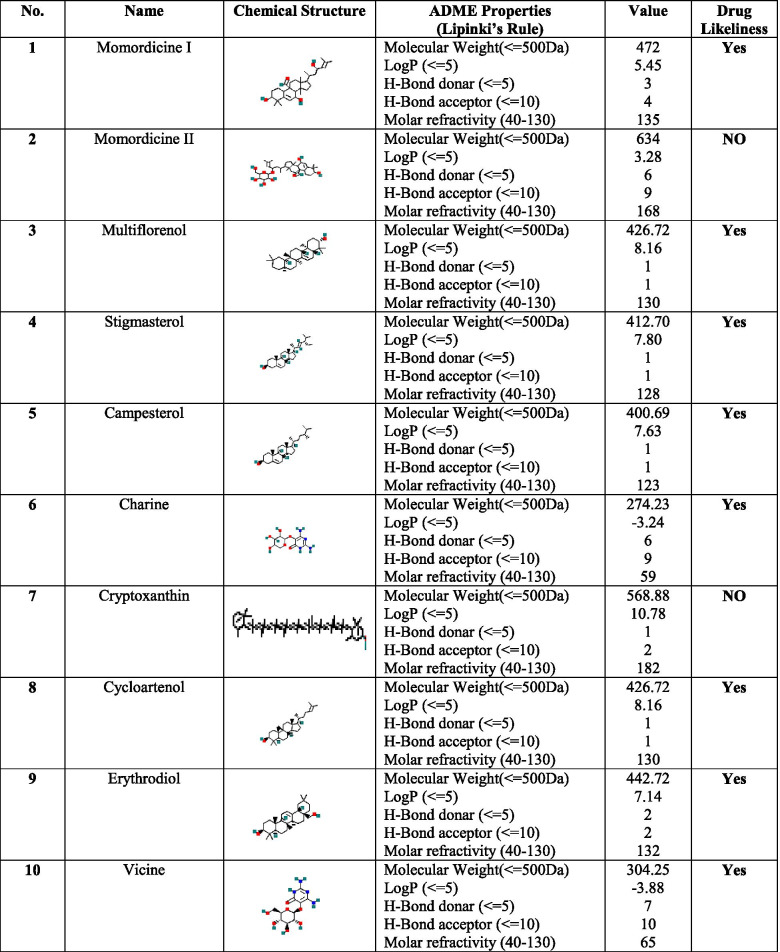
ADME Properties of selected bitter melon (*Momordica charantia*) phytoconstituents against SARS-CoV-2 spike protein

### Identification of the erythrodiol and SARS-CoV-2 spike protein S2 interaction

**Fig. 4:**
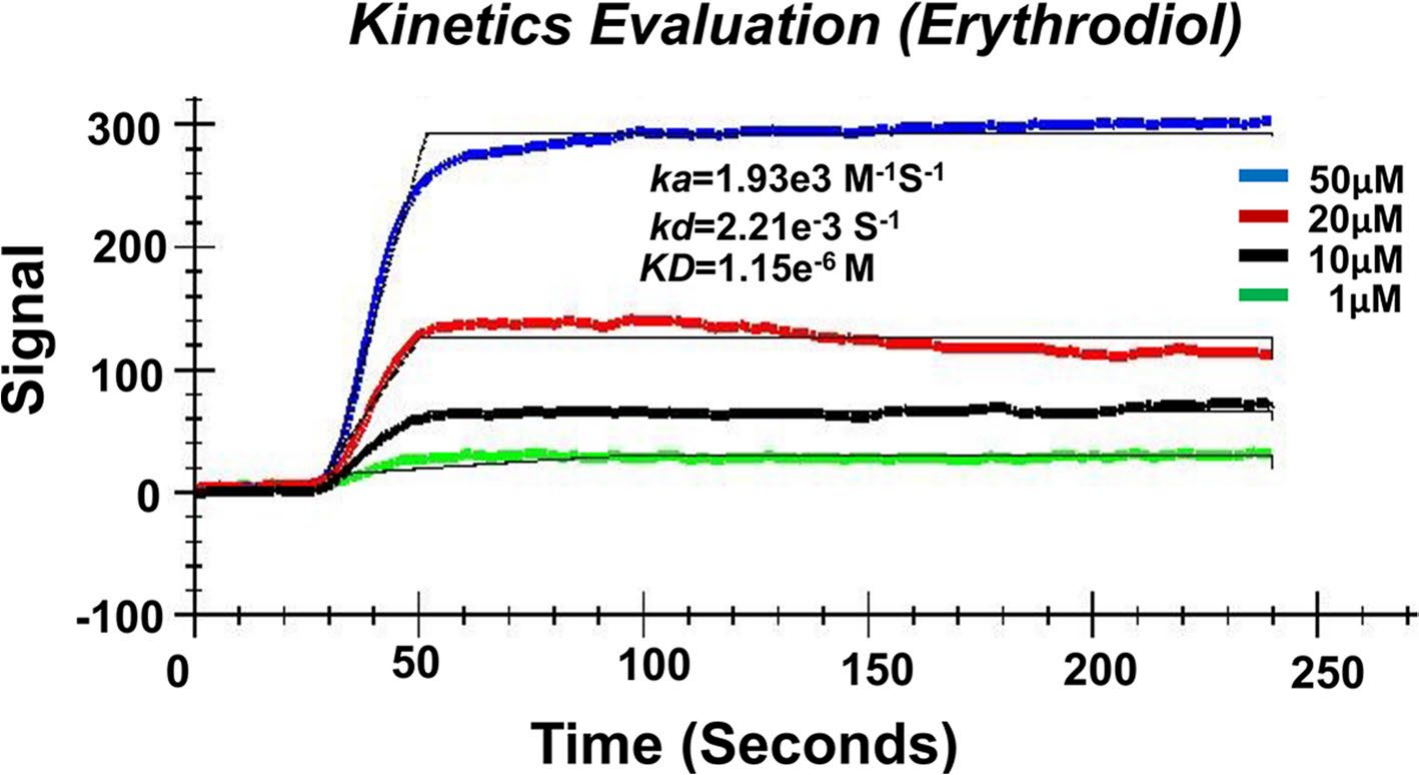
Binding kinetics analysis of SARS-CoV-2 spike protein and the bitter melon phytoconstituent, erythrodiol. Surface plasmon resonance (SPR) sensogram showing the binding kinetics for immobilized SARS-CoV-2 spike protein S2 and the bitter melon phytoconstituent, erythrodiol. A typical response curve at different analyte concentrations (green, 1 μM; black, 10 μM; red, 20 μM; blue, 50 μM) shows the association (ka = 1.93 × 103 M-1S-1) and dissociation (kd = 2.21 × 10 − 3 S-1) rate constant of interaction. A binding affinity between SARS-CoV-2 spike protein S2 and erythrodiol showed an equilibrium dissociation constant (KD) value, 1.15 μM. Data sets were analyzed by one-to-one (1:1) binding fit models using TraceDrawer evaluation software

Assessment of the binding kinetics and affinities between molecules is a well-established practice in drug development. Among advances in technology related to drug and receptor interaction, surface plasmon resonance (SPR) is a label-free technique that can monitor the real-time the association (*k*_on_) and dissociation (*k*_off_) rate constant) molecular interactions. SPR is also used as a tool in a wide variety of interactions of biomolecules, including small molecules, nucleic acids, carbohydrates, lipids, ligand-receptor kinetics, antibody-antigen interactions, enzyme–substrate reactions, and epitope mapping [27–30]. To confirm the binding affinity between erythrodiol and the S2 spike protein, we employed COOH sensor chip-based SPR. A typical SPR signal from experimental data at various analyte concentrations is shown in Fig. 4. Our results showed that the S2 spike protein bound to erythrodiol with a high association rate constant, ka = 1.93 × 10^3^ M^−1^S^−1^; the dissociation rate constant was kd = 2.21 × 10^–3^ S^−1^. The binding kinetics showed an equilibrium dissociation constant (KD) value of 1.15 μM. In sum, erythrodiol displayed a strong interaction with the SARS-CoV-2 spike S2 protein.

## Discussion

COVID-19 outbreaks are threats to human beings across the globe. Scientists, clinicians, and researchers are working to develop effective treatments for the highly contagious coronavirus strains. To date, several compounds are being screened for potency and selectivity before conducting human trials. In computer-aided drug design, particularly for identifying a lead compound [31, 32], molecular docking is employed to explore the various types of binding interactions of prospective drugs with various domains or active sites of the target molecules. The interactions include H-bond, π-π, and amide-π interactions. The binding affinity of a ligand with an active site has been determined by evaluating its hydrogen-bonding pattern [31, 33] and the nature of residues present at the active site. The binding energy (Kcal/mol) data allow comparisons of the binding affinity of various ligands/compounds with their corresponding target receptor molecules. A lower binding energy indicates a higher affinity of the ligand to the receptor. Ligands with high affinities can be selected as potential drugs.

Natural compounds derived from plant extracts are assets in identifying new drugs. To date, only a few natural compounds have been identified as therapeutic with specificity for cellular and molecular targets. For instance, Taxol binds to β-tubulin, which distorts the cytoskeleton framework and causes cell-cycle arrest and, subsequently, cell death, has been used for several decades to treat various tumors [34]. Furthermore, natural compounds exhibit a myriad of biological activities, including antiviral activity. A virtual screening study demonstrates that natural compounds can inhibit the binding of SARS-CoV-2 to ACE2 [35]. Here, we investigated the effect of biomolecule extracted from *Momordica charantia* to block the SARS-CoV-2 spike protein, which is necessary for SARS-CoV-2 infection. Components from *Momordica charantia* exhibit immunosuppressive as well as immunostimulatory activity. The immunosuppressive activity of alpha- and beta-momorcharin is due to lymphocytotoxicity or to a shift in the kinetic parameters of the immune response [36]. In contrast to this, other studies have shown that this compound potentiates the immune system by increasing natural killer cell numbers and cytotoxic activity [37]. At doses of 25, 50, and 100 mg/kg body weight, an ethanolic extract showed a stimulatory effect on both humoral and cellular functions in vivo [38]. Furthermore, the immunomodulatory activity of *Momordica charantia* fruits and seeds is attributed to their property of inhibiting the release of TNF-α and nitric oxide (NO) [38].

Erythrodiol, a precursor of pentacyclic triterpenic acids*,* displays activity against HIV-1 reverse transcriptase with an IC50 of 5 μM [24]*.* To characterize the binding interaction between erythrodiol and SARS-CoV-2 spike protein S2, we employed SPR, which has been used to screen interactions between potential drugs and their receptors. Our results demonstrate that erythrodiol has high selectively (binding affinity 1.15 μM) and inhibitory activity for SARS-CoV-2 spike protein S2. This is supported by the fact that SPR technology has been used to screen a library of 960 compounds for binding to ACE2 [5].

## Conclusions

An *in-silico* approach to finding a natural compound that binds and prevents the attachment/internalization of the SARS-CoV-2 virus is a therapeutic and preventive option for the development of drugs with a time constraint. Bioinformatics approaches to make a fast and more or less accurate predictions for potential drugs or inhibitors. In this study, we used multiple bioinformatics tools to identify potent natural compounds, mainly flavonoids, that target and bind to the spike protein of SARS-CoV-2. We identified 10 flavonoids capable of binding either to the S1 or S2 domain of the SARS-CoV-2 protein. Our findings suggest that compounds from *Momordica charantia* have the potential to inhibit the SARS-CoV-2 spike protein and should be explored further as agents for preventing COVID-19.

## Materials and methods

### Ligands and Receptor

The 3-dimensional structures of phytoconstituents of bitter melon (*Momordica charantia*) were downloaded from the PubChem database, and these structures were converted to a Protein Data Bank (PDB) format by use of chimera software. The structure of SARS-CoV-2 S (spike protein) (Fig. 1) was downloaded from the RCSB protein data bank (PDB-ID: 6VYB) [8]. The structures of the ligands are provided in Table 1.

### Molecular docking of bitter melon (*Momordica charantia*) phytoconstituents to the SARS-CoV-2 spike protein

The structure of SARS-CoV-2 spike protein was used for the docking analysis. SARS-CoV-2 spike protein is a heterotrimer consisting of chains A, B, and C [8]. For the docking experiment, chain A of the spike protein was used. All the docking experiment was performed using MTiOpenScreen web server. First, the SARS-CoV-2 spike protein file was uploaded with respective bitter melon (*Momordica charantia)* phytoconstituents, and then virtual screening was performed using AutoDock Vina [39], with employs a gradient-based conformational search. AutoDock defines the search space by a grid box defined by the box center coordinates with its dimensions of x, y, and z. In AutoDock Vina, the grid resolution is internally assigned to 1 Å. We used the number of binding modes of 10 and exhaustiveness of 8. The grid dimensions and center provided were automatically calculated based on the protein residues of the binding site. The scoring of the generated docking poses and ranking of the ligands were based on the Vina empirical scoring function and approximated the kcal/mol's binding affinity. The hydrophilic and hydrophobic interactions were determined using PyMol and Schrodinger software.

### Prediction of ADME analysis

ADME profiling of the phytoconstituents was determined using online software tools at pH 7 [25]. The essential parameters allied with ADME properties, such as Lipinski’s rule of five and the drug's solubility, pharmacokinetic properties, molar refractivity, and likeliness, were deliberated [26]. All calculated values are shown in Table 1.

### Surface plasmon resonance (SPR) signal detection in SARS-CoV-2 spike protein and erythrodiol binding

Following our previous publication [40], we investigated the binding interaction between SARS-CoV-2 spike protein and erythrodiol using an OpenSPR instrument (Nicoya Lifescience, ON, Canada). Briefly, for covalent coupling, the first carboxyl (COOH) sensor chip was loaded into the instrument and pumped with running buffer 1X PBS (pH 7.4). Next, the amine coupling kit EDC:1-(3-dimethylaminopropyl)-3-ethylcarbodiimide hydrochloride; NHS: N-hydroxysuccinimide (EDC/NHS) was applied as described in the manufacturer’s protocol. Further, a ligand, recombinant protein SARS-CoV-2 spike protein S2 (50 μg/mL, Fisher Scientific, PA, USA) was diluted in activation buffer and immobilized on a sensor chip. After 5 min of interaction, it was blocked with a blocking buffer supplied by the manufacturer (Nicoya Lifescience, ON, Canada), followed by the blank buffer. Subsequently, various concentrations (1 μM-50 μM) of analyte erythrodiol (Fisher Scientific, PA, USA) were injected onto the ligand-immobilized sensor chip with a flow rate of 20 μL/min. Further, to assess the interaction, the buffer blank generated by the flow cell was subtracted, and the data sets were analyzed by one-to-one (1:1) binding fit models using Trace Drawer software.

## Data Availability

All datasets analyzed during this study are included in the article.

## Abbreviations

SARS-CoV-2: Severe acute respiratory syndrome coronavirus 2
ACE 2: Angiotensin-converting enzyme 2
RBD: Receptor-binding domain
HIV: Human immunodeficiency virus
ADME: Absorption, distribution, metabolism, and excretion
SPR: Surface plasmon resonance
KD: Equilibrium dissociation constant
TNF: Tumor necrosis factor
PDB: Protein data bank
RCSB PDB: Research Collaboratory for Structural Bioinformatics Protein Data Bank
